# The PDGF-D/miR-106a/Twist1 pathway orchestrates epithelial-mesenchymal transition in gemcitabine resistance hepatoma cells

**DOI:** 10.18632/oncotarget.3193

**Published:** 2015-02-11

**Authors:** Rui Wang, Yumei Li, Yueyue Hou, Qingling Yang, Sulian Chen, Xi Wang, Zishu Wang, Yan Yang, Changjie Chen, Zhiwei Wang, Qiong Wu

**Affiliations:** ^1^ Department of Medical Oncology, First Affiliated Hospital of Bengbu Medical College, Bengbu, Anhui, China; ^2^ Department of Biochemistry and Molecular Biology, Bengbu Medical College, Anhui, China; ^3^ Department of Oncology, The 117th Hospital of PLA, Hangzhou, China; ^4^ The Cyrus Tang Hematology Center and Collaborative Innovation Center of Hematology, Jiangsu Institute of Hematology, the First Affiliated Hospital, Soochow University, Suzhou, China

**Keywords:** Hepatocellular carcinoma, EMT, PDGF-D, miR-106a, Twist

## Abstract

Emerging evidence demonstrates that platelet-derived growth factor-D (PDGF-D) plays a critical role in epithelial-mesenchymal transition (EMT) and drug resistance in hepatocellular carcinoma (HCC) cells. However, the underlying mechanism has not been fully elucidated. The objective is to explore the molecular mechanism of PDGF-D-mediated EMT in drug resistance HCC cells. To achieve our goal, we used multiple approaches including Western blotting, real-time RT-PCR, wound healing assay, invasion assay, luciferase activity assay, transfection, and immunohistochemistry. We found that PDGF-D is highly expressed in gemcitabine-resistant (GR) HCC cells. Moreover, PDGF-D markedly inhibited miR-106a expression and subsequently upregulated Twist1 expression. Notably, PDGF-D expression was associated with miR-106a and Twist1 in HCC patients. Our findings provide a possible molecular mechanism for understanding GR chemoresistance in HCC cells. Therefore, inactivation of PDGF-D/Twist or activation of miR-106a could be a novel strategy for the treatment of HCC.

## INTRODUCTION

Hepatocellular carcinoma (HCC) is one of commonly diagnosed malignancies [1]. Sorafenib, the standard of care for patients with advanced HCC, improved the median survival in advanced HCC; however, the median overall survival is less than one year [2, 3]. Several other drugs targeting signaling cascades such as brivanib, sunitinib, erlotinib, and linifanib encountered setbacks in clinical trials in advanced HCC [4, 5]. Recently, infusional fluorouracil, leucovorin, and oxaliplatin (FOLFOX4) as palliative chemotherapy to patients with advanced HCC conferred some benefit [6]. Moreover, it has been shown that gemcitabine and oxaliplatin are effective with manageable toxicity in advanced HCC patients [7]. However, tumor cells acquire resistance to these chemotherapeutic drugs, leading to treatment failure and high mortality of HCC [8].

A line of evidence demonstrates that chemo-resistance is associated with the acquisition of epithelial-mesenchymal transition (EMT) of cancer cells, leading to enhanced metastasis [9]. Our previous studies have demonstrated that chemotherapeutic drug gemcitabine-resistant (GR) HCC cells acquired EMT characteristics [10]. It is known that during EMT, epithelial cells convert into mesenchymal cells through losing epithelial cell-cell junction and epithelial markers such as E-cadherin and γ-catenin, and gaining the expression of mesenchymal markers such as Twist, Vimentin, Snail, Slug, which lead to increased migration and invasion. Notably, we identified that platelet-derived growth factor-D (PDGF-D) signaling pathway plays a critical role in the acquisition of EMT phenotype of GR HCC cells [10]. Although PDGF-D is critically involved in tumorigenesis [11], the exact mechanism by which PDGF-D regulates EMT in GR cells has not been fully elucidated. Therefore, identifying the underlying mechanism of PDGF-D-mediated EMT could be helpful to find novel strategy to treat HCC patients.

Emerging evidence has suggested that microRNAs (miRNAs) play an important role in regulation of EMT in HCC. It has been accepted that miRNAs elicit their regulatory effects by binding to the 3′ untranslated region (3′UTR) of target mRNA, resulting in the degradation of the mRNA or translational inhibition of functional proteins. The growing body of literature strongly suggests the essential roles of miRNAs in EMT progresses in HCC. Zhang et al. reported that miR-490-3p modulates EMT by targeting endoplasmic reticulum-Golgi intermediate compartment protein 3 (ERGIC3) in HCC cells [12]. Similarly, overexpression of miR-216a/217 induced EMT through activation of PI3K (phosphatidylinositol 3-kinase)/Akt and TGF-β (transforming growth factor, beta 1) pathways by targeting PTEN (phosphatase and tensin homolog deleted on chromosome ten) and SMAD7 (SMAD family member 7), leading to contribution to tumor recurrence in HCC [13]. Consistently, miR-612 was found to suppress EMT through regulation of Akt2 in HCC [14]. Moreover, miR-612 inhibited the stemness of HCC through targeting Wnt/β-catenin signaling [15]. Furthermore, overexpression of miR-106b triggered EMT and subsequently promoted cell migration and metastasis in HCC [16]. These studies imply that miRNAs govern EMT through regulation of their targets.

Recent studies have revealed that PDGF-D crosstalks with miRNA [17, 18]. Specifically, PDGF-D overexpression let to the acquisition of EMT phenotype in prostate cancer cells consistent with the loss of miR-200 expression, indicating that PDGF-D-induced EMT is in part due to down-regulation of miR-200 expression [17]. The goal of this study was to determine whether PDGF-D mediated EMT through regulation of miRNAs in HCC GR cells. In the current study, we reported that PDGF-D/miR-106a/Twist pathway orchestrates EMT in HCC GR cells. Our results further suggest that targeting PDGF-D could be a novel strategy for the treatment of HCC patients.

## RESULTS

### HCC GR cells have EMT characteristics

**Figure 1:**
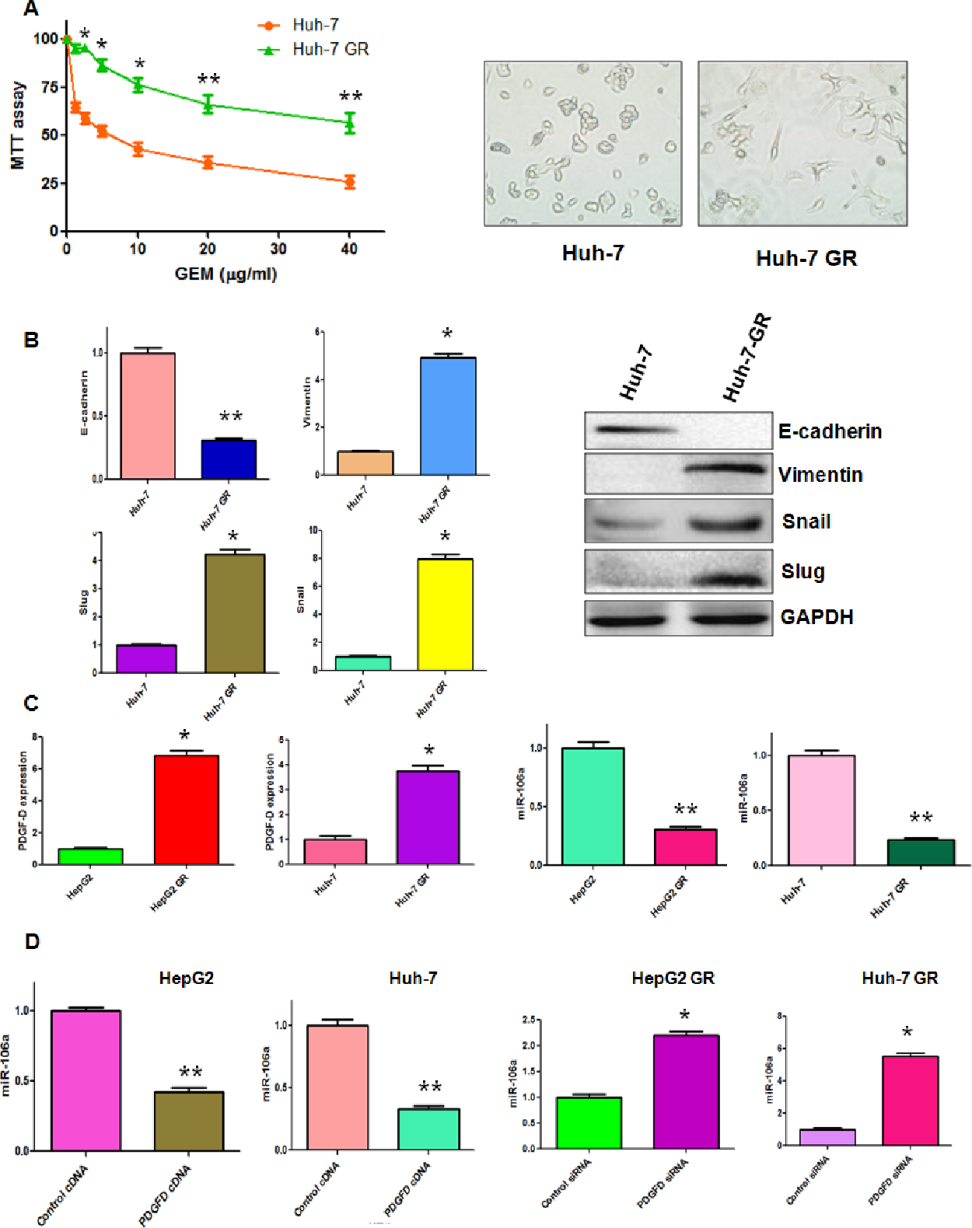
PDGF-D regulates miR-106a expression **(A)** Left panel: MTT assay was performed in parental Huh-7 and Huh-7 GR cells, respectively. Right panel: The morphology of Huh-7 and Huh-7 GR cells was observed by microscopy. *p < 0.05, **p < 0.01 GR vs control. **(B)** Left panel: Real-time RT-PCR assay was performed to detect the mRNA levels of E-cadherin, Slug, Snail, and Vimentin in Huh-7 and Huh-7 GR cells. *p < 0.05, **p < 0.01 vs control. Right panel: Western blotting analysis was conducted to measure the expression of E-cadherin, Snail, Slug, and Vimentin in Huh-7 and Huh-7 GR cells. **(C)** Left panel: Real-time RT-PCR assay was performed to detect the mRNA level of PDGF-D in HepG2, HepG2 GR, Huh-7 and Huh-7 GR cells. Right panel: miR-106a was measured by miRNA real-time RT-PCR in indicated HCC cells. *p < 0.05, **p < 0.01 vs control. **(D)** miR-106a was measured by miRNA real-time RT-PCR in indicated HCC cells after modulation of PDGF-D. *p < 0.05, **p < 0.01 vs control.

Our previous study has shown that HepG2 GR cells have EMT phenotype [10]. To further determine whether Huh-7 GR cells could acquire EMT feature, we developed Huh-7 GR cells (Figure 1A). As expected, we observed that Huh-7 GR cells, like HepG2 GR cells, had morphologic changes consistent with EMT. Huh-7 GR cells displayed elongated fibroblastic morphology compared with Huh-7 parental cells with epithelial cobblestone phenotype (Figure 1A). More importantly, our RT-PCR and Western blotting analysis revealed that Huh-7 GR cells have low expression of E-cadherin and high expression of Vimentin, Snail and Slug, suggesting that Huh-7 GR cells obtained the specific EMT molecular markers (Figure 1B).

### Down-regulation of miR-106a in HCC GR cells

PDGF-D has been reported to be critically involved in GR-mediated EMT [11], we measured the expression of PDGF-D at mRNA and protein levels in HCC GR cells by RT-PCR and Western blotting, respectively. Consistent with our previous report [10], we observed a significantly increased PDGF-D at both mRNA and protein levels in HepG2 GR and Huh-7 GR cells (Figures 1C, 5B). It has been known that miR-106a plays a pivotal role in drug resistance [19]. Thus, we determine whether miR-106a has changes in HCC GR cells compared with the parental cells. Indeed, we observed the down-regulation of miR-106a in both HepG2 GR and Huh-7 GR cells (Figure 1C).

### PDGF-D regulates miR-106 expression

Next, we explored whether PDGF-D could regulate the expression of miR-106a in HCC cells. To address this question, we depleted PDGF-D by its specific siRNA in HCC GR cells and up-regulated PDGF-D expression using its cDNA plasmid in HCC cells. We observed that PDGF-D siRNA significantly inhibited the expression of PDGF-D in HCC GR cells, whereas PDGF-D cDNA remarkably increased PDGF-D expression in HCC cells (data not shown). Moreover, we found that miR-106a was inhibited by PDGF-D cDNA transfection in HepG2 and Huh-7 cells (Figure 1D). On the contrary, miR-106a was increased in HepG2 GR and Huh-7 GR cells after depletion of PDGF-D (Figure 1D).

### miR-106a inhibits cell migratory and invasive activity in GR cells

**Figure 2:**
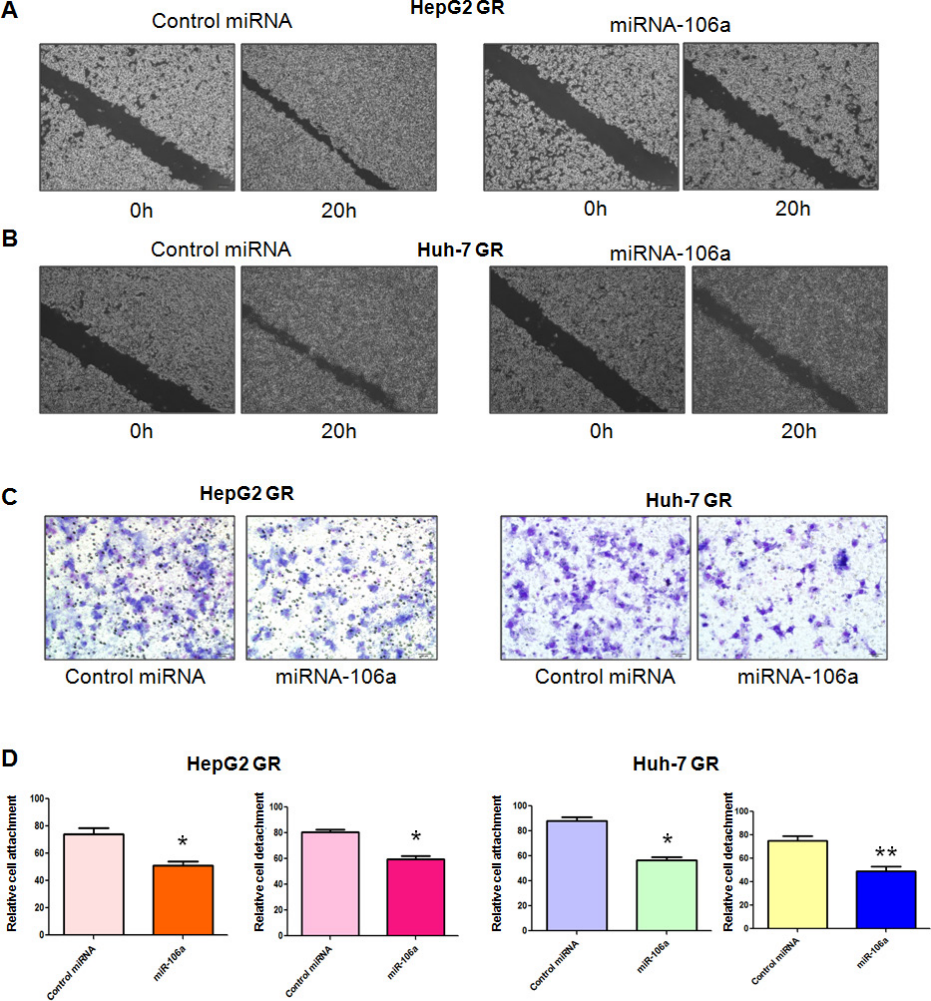
miR-106a inhibits cell migratory and invasive activity **(A–B)** Wound assays were performed to compare the migratory potential of HepG2 GR (A) and Huh-7 GR (B) cells after miR-106a treatment. **(C)** Invasion assay was conducted to measure the invasive capacity in HepG2 GR and Huh-7 GR cells after miR-106 treatment. **(D)** Cell attachment and detachment assays were conducted in HepG2 GR and Huh-7 GR cells after miR-106 treatment. *P < 0.05, **p < 0.01 vs control.

It has been accepted that cells obtain enhanced migration and invasion during EMT. To validate the role of miR-106a in regulation of EMT, we conducted the wound-healing assay and invasion assay in HCC GR cells treated with miR-106a mimic. As demonstrated in Figure 2A and 2B, HepG2 GR and Huh-7 GR cells with miR-106a treatment have significantly decreased numbers of cells migrating across the wound. Moreover, miR-106a mimic inhibited cell invasion in HCC GR cells compared with control miRNA treatment (Figure 2C). Furthermore, we found that miR-106a treatment suppressed the cell attachment and detachment in both HepG2 GR and Huh-7 GR cells (Figure 2D).

### Twist1 is a downstream target of miR-106a

**Figure 3:**
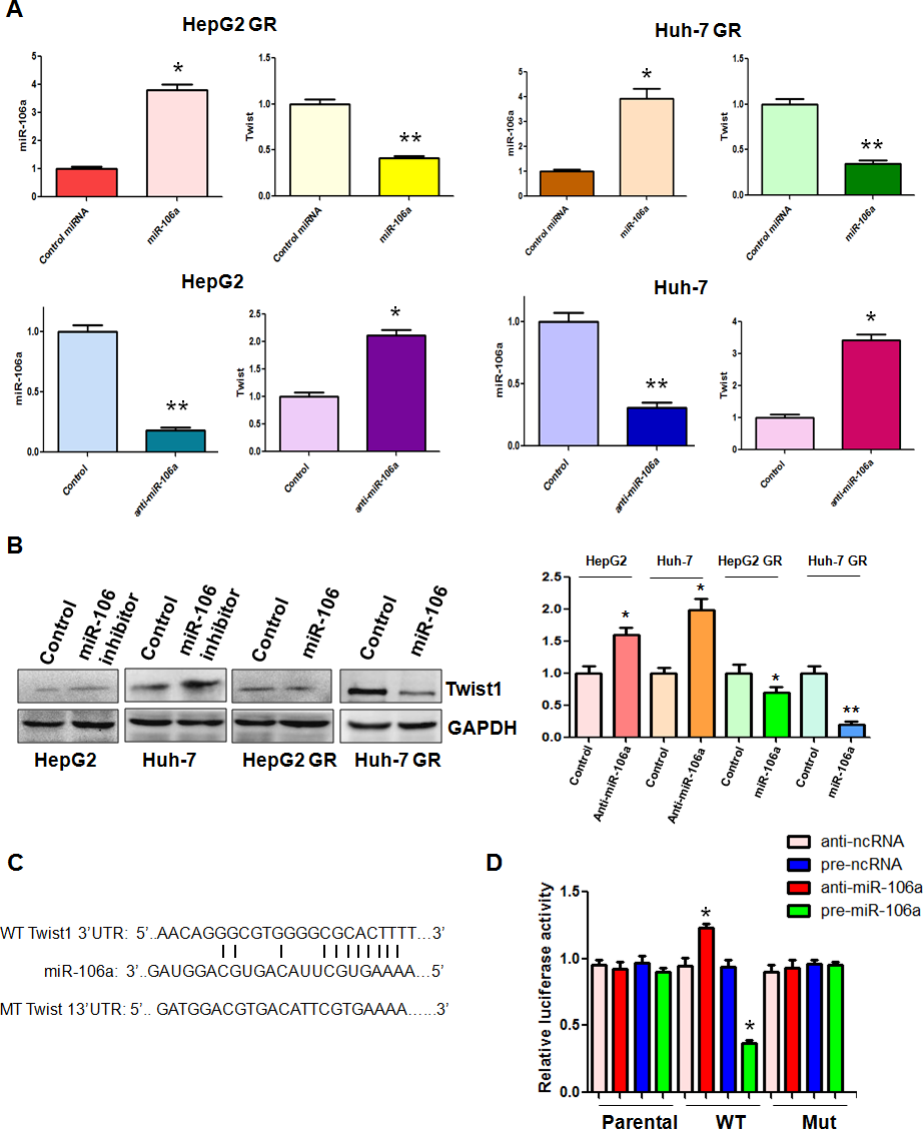
miR-106a regulates Twist1 expression **(A)** Top panel: Real-time RT-PCR assay was performed to detect the mRNA level of Twist1 in HCC GR cells treated with miR-106a mimics. miR-106a was measured by miRNA real-time RT-PCR in HCC GR cells after miR-106a mimic transfection. *p < 0.05, **p < 0.01 vs control. Bottom panel: Real-time RT-PCR assay was performed to detect the mRNA level of Twist1 in HCC cells treated with miR-106a inhibitor. miR-106a was measured by miRNA real-time RT-PCR in HCC cells after miR-106a inhibitor treatment. *p < 0.05, **p < 0.01 vs control. **(B)** Left panel: Western blotting analysis was conducted to measure the expression of Twist1 in HCC cells treated with miR-106a inhibitor and in HCC GR cells treated with miR-106a mimic. Right panel: Quantitative results are illustrated for left panel. **(C)** Sequences of wild-type and mutant target sites for miR-106a in Twist1 are shown. **(D)** Luciferase reporter assays were performed to identify the binding of miR-106a to Twist1 3′-UTR. WT: wild type; Mut: mutation. *p < 0.05, **p < 0.01 vs control.

To further explore the molecular mechanism of miR-106a-regulated EMT in HCC GR cells, we sought to identify the target of miR-106a. According to the data from three public algorithms including TargetScan, PicTar, and miRanda, Twist1 could be a potential target of miR-106a. Since Twist plays an essential role in EMT, we focused our study on Twist1. Our results from RT-PCR demonstrated that miR-106a mimic treatment caused down-regulation of Twist1 in HCC GR cells, whereas miR-106a inhibitor treatment led to up-regulation of Twist1 in HCC cells (Figure 3A). Western blotting analysis also showed that increased level of Twist1 was observed in HCC cells after miR-106 inhibitor treatment (Figure 3B). Consistently, a reduced level of Twist1 was found in HCC GR cells treated with miR-106a mimic (Figure 3C). Further bioinformatics analysis indicated that the Twist1 3′UTR harbors potential miR-106a target sites (Figure 3C). To further verify the Twist1 as a target of miR-106a, we performed reporter assays in HCC cells with the luciferase gene driven by either wild-type or mutated Twist1 3′UTR sequences (Figure 3C). Our results showed that it has a significant reduction in luciferase activity with wild-type Twist1, but not mutant Twist1, in HepG2 GR cells transfected with miR-106a mimic (Figure 3D). In line with this, treatment with miR-106a inhibitor resulted in increased luciferase activity with wild-type Twist1 in HepG2 cells (Figure 3D). Altogether, these findings support that miR-106a binds to Twist1 and that Twist1 is a downstream target of miR-106a.

### Down-regulation of Twist1 reverses EMT to MET in GR cells

**Figure 4:**
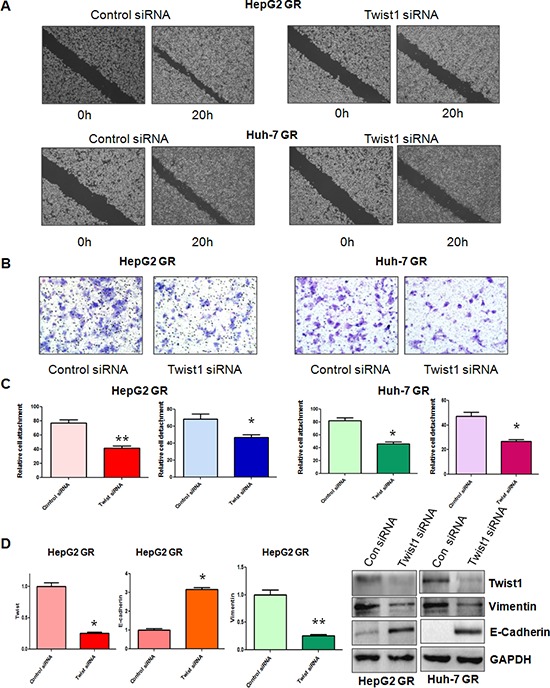
Down-regulation of Twist1 reverses EMT in GR cells **(A)** Wound assays were performed to compare the migratory potential of HepG2 GR (Top panel) and Huh-7 GR (Bottom panel) cells after Twist1 siRNA transfection. **(B)** Invasion assay was conducted to measure the invasive capacity in HepG2 GR and Huh-7 GR cells after Twist1 siRNA transfection. **(C)** Cell attachment and detachment assays were conducted in HepG2 GR and Huh-7 GR cells after Twist1 siRNA transfection. *p < 0.05, **p < 0.01 vs control. **(D)** Left panel: Real-time RT-PCR assay was performed to detect the mRNA level of Twist1, E-cadherin, Vimentin in HepG2 GR cells treated with Twist1 siRNA. *p < 0.05, **p < 0.01 vs control. Right panel: Western blotting analysis was conducted to measure the expression of Vimentin, and E-cadherin in HCC GR cells treated with Twist1 siRNA.

To determine whether Twist1 is involved in GR-mediated EMT, we depleted the Twist1 using its specific siRNA in HepG2 GR and Huh-7 GR cells. Then, we performed the wound-healing assay and invasion assay in HCC GR cells transfected with Twist1 siRNA. The wound-healing assay showed that depletion of Twist1 inhibited cell migration in HCC GR cells (Figure 4A). In support of this note, we also found that Twist1 siRNA treatment led to inhibition of cell invasion (Figure 4B). Notably, our results demonstrated that depletion of Twist1 suppressed the cell detachment and attachment in HCC GR cells (Figure 4C). Importantly, we observed that HepG2 GR and Huh-7 GR cells transfected with Twist1 siRNA displayed round cell-like morphology (data not shown). More importantly, our RT-PCR results revealed that down-regulation of Twist1 decreased E-cadherin mRNA, but increased Vimentin mRNA in HepG2 GR (Figure 4D) and Huh-7 GR (data not shown). The Western blot analysis also confirmed that the expression of E-cadherin was increased, while Vimentin expression was decreased in HCC GR cells with Twist1 siRNA treatment (Figure 4D). These results indicated that depletion of Twist1 led to the reversal of EMT to MET phenotype.

### PDGF-D controls Twist1 expression

**Figure 5:**
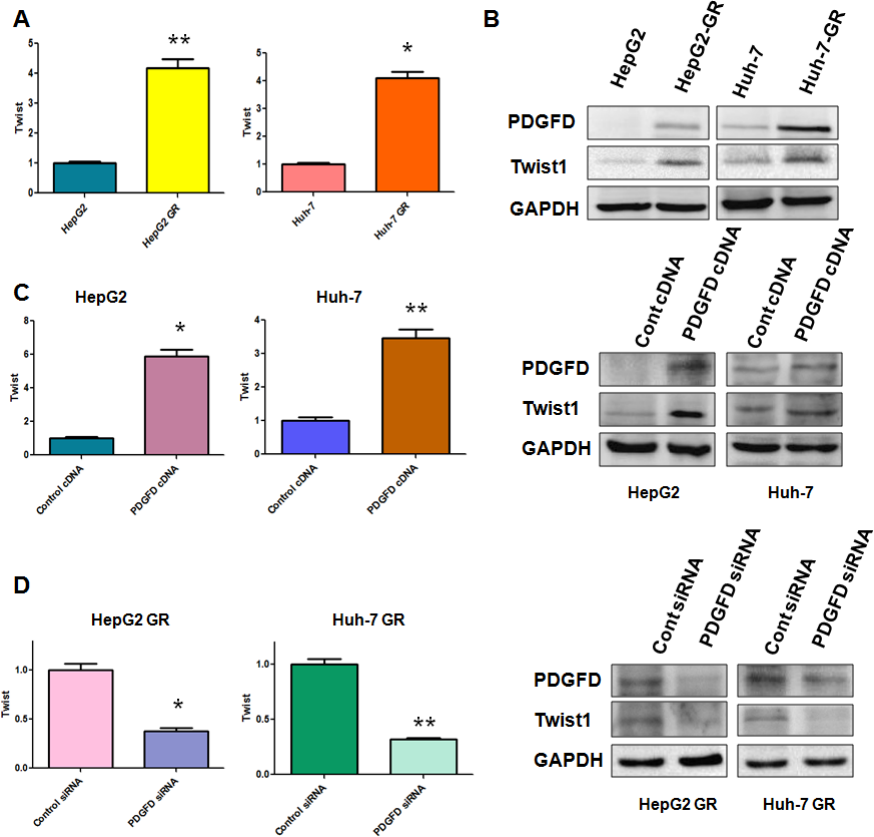
PDGF-D controls Twist1 expression **(A)** RT-PCR assay was performed to detect the mRNA level of Twist1 in indicated HCC cells. *p < 0.05, **p < 0.01 vs control. **(B)** Western blotting analysis was conducted to measure the expression of Twist1, PDGF-D in indicated HCC cells. **(C)** RT-PCR assay and Western blotting analysis were used to detect the expression of Twist1 in HepG2 and Huh-7 cells after PDGF-D cDNA transfection. *p < 0.05, **p < 0.01 vs control. **(D)** RT-PCR assay and Western blotting analysis were used to detect the expression of Twist1 in HepG2 GR and Huh-7 GR cells after PDGF-D siRNA transfection. *p < 0.05, **p < 0.01 vs control.

We have found that PDGF-D regulates miR-106a expression and Twist1 is a target of miR-106a. Thus, we investigated whether PDGF-D controls Twist1 expression. As we expected, increased Twist1 expression at mRNA and protein levels were observed in HCC GR cells, which have high expression of PDGF-D compared with parental HCC cells (Figure 5A, 5B). In line with this finding, we found that over-expression of PDGF-D up-regulated Twist1 expression in HCC cells (Figure 5C). In contrast, depletion of PDGF-D down-regulated Twist1 expression in HCC GR cells (Figure 5D). Taken together, our results suggest that PDGF-D could govern Twist1 expression.

### PDGF-D expression is associated with miR-106a and Twist1 in HCC patients

**Figure 6:**
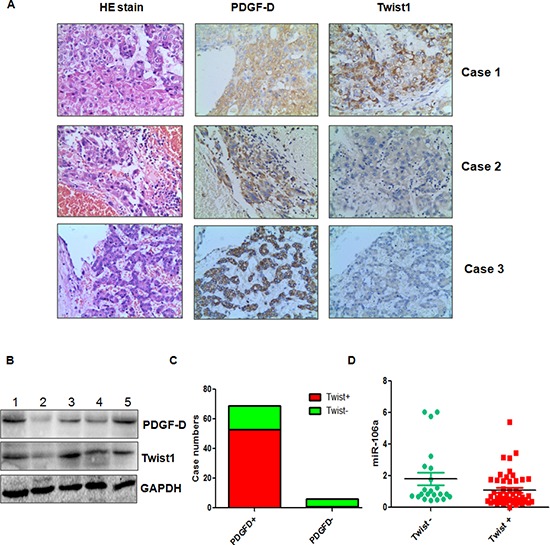
PDGF-D is associated with miR-106a and Twist1 in HCC tissues **(A)** Three representative cases are shown that PDGF-D is correlated with Twist1 in HCC tissues. **(B)** The expression of PDGF-D and Twist1 was determined by Western blotting in HCC clinical tissues. **(C)** The percentage of specimens showing high PDGF-D expression in relation to the expression of Twist1. **(D)** miR-106a is inversely correlated with Twist1 in HCC tissues. *p < 0.05 Twist1 positive vs Twist1 negative.

To further define the physiological function of PDGF-D and Twist1 in HCC, we measured their expressions using immunohistochemical staining in 76 HCC patients. We found that sixty-nine of 76 (90%) HCC were positive for PDGF-D protein expression, while 7 (10%) of the cases were negative (Figure 6A, 6C). We also observed that 54 (71%) HCC patients had Twist1 expression. More importantly, PDGF-D expression was significantly associated with Twist1 expression (Figure 6A, 6C). To investigate whether Twist1 was associated with miR-106a expression in HCC patients, we detected the miR-106 level in 76 HCC samples. We found a significant association between Twist1 and miR-106a expression (Figure 6D). Twist1 positive samples had low expression of miR-106a, whereas Twist1 negative samples obtained high expression of miR-106a (Figure 6D). To further validate the relationship between PDGF-D and Twist1 expression in HCC, we randomly selected the 20 HCC tissues and detected the PDGF-D and Twist1 levels by Western blotting analysis. Consistent with the results from immunohistochemical staining, we observed a correlation between PDGF-D and Twist1 in HCC samples (Figure 6B). These findings indicated the tight regulatory relationships among PDGF-D, Twist1, and miR-106a in HCC.

## DISCUSSION

The data presented here demonstrated that PDGF-D mediated EMT through inhibition of miR-106a and subsequent upregulation of Twist1 in HCC GR cells. Recent studies have identified critical roles of miRNAs in EMT processes. For example, miR-21 induced EMT due to targeting both human sulfatase-1 and PTEN and subsequent activation of Akt/Erk (extracellular regulated protein kinase) pathways in HCC cells [20]. Similarly, miR-491 inhibited EMT via modulation of matrix metalloproteinase 2/9 in HCC cells [21]. In addition, miR-29a regulated TGF-β-induced EMT through the inhibition of DNA methyltransferases in HCC cells [22]. Moreover, it has been reported that miR-148a suppressed EMT and cancer stem cells-like properties by targeting Wnt1 (wingless-type MMTV integration site family member 1) and Met, leading to inhibition of metastasis of HCC [23, 24]. Furthermore, miR-331-3p promotes EMT-mediated metastasis of HCC through the inhibition of PHLPP (PH domain and leucine rich repeat protein phosphatase)-mediated dephosphorylation of Akt [25]. In support of role of miRNAs in EMT, miR-26b was found to suppress EMT through targeting USP9X (ubiquitin specific peptidase 9, X-linked) in HCC [26]. Recently, one study revealed that miR-106a was implicated in ovarian carcinoma-associated EMT [27]. In line with these findings, we observed that miR-106a was increased in EMT-type GR cells. Notably, miR-106a inhibited cell migratory and invasive activity in GR cells. Our study provided a new mechanism by which PDGF-D regulated EMT through inhibition of miR-106a. It is noteworthy that PDGF-D mediated EMT in prostate cancer cells partly through repressing miR-200b [17]. Interestingly, we did not observe any changes in miR-200b level in GR HCC cells (data not shown), suggesting that miR-200b is not involved in GR-mediated EMT in HCC cells.

Twist family has Twist1 and Twist2, which are two distinct tissue-restricted transcription factors with high sequence similarity. Twist family is critically involved in governing EMT process [28]. It has been known that Twist inhibited E-cadherin and triggered an EMT, leading to tumor invasion and metastasis [29]. Consistently, higher expression of Twist is correlated with tumor invasion and metastasis in breast cancer [29]. Moreover, Twist was reported as a prognostic biomarker in certain human cancers [28]. Strikingly, Twist has been found to be critically involved in drug resistance. For example, knockdown of Twist1 enhanced cell death induced by arsenic trioxide- and ionizing radiation in lung cancer cells [30]. Similarly, Twist1 knockdown sensitized prostate cancer cells to docetaxel treatment [31]. In keeping with this, Twist confers chemoresistance to anthracyclines by uoregulation of P-glycoprotein in bladder cancer cells [32]. Multiple studies have identified that some factors and signaling pathways including HIF-1 (hypoxia inducible factor-1) [33], SRC-1 (steroid receptor coactivator-1) [34], STAT3 (signal transducer and activator of transcription 3) [35, 36], F-box protein FBXL14 (F-box and leucine-rich repeat protein 14) [37], β-TRCP (beta-transducin repeat-containing protein) [38], and Notch1 [39] govern Twist expression. Strikingly, accumulating evidence suggests that miRNAs regulate Twist expression. For instance, down-regulation of miR-214 promoted EMT by directly targeting Twist gene in intrahepatic cholangiocarcinoma [40]. Long et al. reported that down-regulation of miR-138 promoted colorectal cancer metastasis through directly targeting Twist2 [41]. Additionally, miR-181a modulated EMT and metastatic potential through targeting Twist1 in tongue squamous cell carcinoma [42]. Consistently, miR-720 suppressed tumor migration and invasion and through targeting Twist1 in breast cancer [43]. Furthermore, miR-106b inhibited EMT by targeting Twist1 in invasive endometrial cancer cell lines [44]. In line with the role of miRNAs in regulating Twist, our study demonstrated that miR-106a inhibited cell invasion through down-regulation of Twist1 in HCC GR cells. We further validated Twist1 as a downstream target of miR-106a. Since down-regulation of Twist1 reversed EMT to MET in GR cells, miR-106a suppressed EMT partly due to targeting Twist1.

Emerging evidence has indicated that PDGF-D plays a pivotal role in drug resistance. Moreover, knockdown of Twist inhibited EMT and led to a reversal of the chemoresistance in tongue squamous cell carcinoma [42]. Multiple studies have implicated that miRNAs have a key role in drug resistance [45]. For example, miR-17-5p was reported to promote chemotherapeutic drug resistance through repressing PTEN expression in colorectal cancer [46]. Zhang et al. found that miR-205 could be a tumor radiosensitizer via targeting ZEB1 and Ubc13 [47]. Similarly, miR-143 was found to enhance temozolomide-induced apoptosis through targeting N-RAS in glioma [48]. Additionally, one study showed that methylation of miR-129-5p CpG island governed multi-drug resistance by targeting ABC transporters in gastric cancer [49]. Recently, miR-106a has been considered as key factors to modulate chemoresistance. For example, upregulation of miR-106a was associated with paclitaxel resistance in ovarian cancer cells [19]. Moreover, miR-106a modulates cisplatin sensitivity through regulation of PDCD4 (programmed cell death 4) in ovarian cancer cells [50]. Furthermore, miR-106a confers cisplatin resistance through targeting ABCA1 (adenosine triphosphatase-binding cassette A1) in NSCLC (non-small cell lung cancer) cells [51]. Similarly, miR-106a promoted chemoresistance of gastric cancer cells and suppressed drug-induced apoptosis through targeting RUNX3 (runt related transcription factor 3) [52]. Importantly, miRNAs were found to modulate chemoresistance via targeting Twist. One study showed that miR-33a promoted osteosarcoma cell resistance to cisplatin via down-regulation of Twist [53]. Another study suggested that miR-181a reversed chemoresistance due to down-regulation of Twist1 in tongue squamous cell carcinoma [42]. Our current study demonstrated that miR-106a regulated Twist1 and reversed EMT, which could modulate chemoresistance in HCC GR cells. Altogether, miR-106a could be a common miRNA for drug resistance-associated EMT in human cancers.

To this end, we found for the first time that PDGF-D markedly inhibited miR-106a expression. Moreover, we observed that miR-106a targeted Twist expression. Furthermore, we observed that miR-106a inhibited EMT in HCC GR cells. More importantly, PDGF-D expression was associated with miR-106a and Twist in HCC patients. However, further in-depth investigation is required to fully elucidate the molecular mechanisms of PDGF-D-mediated EMT in HCC GR cells. In summary, our results provide a possible molecular mechanism for the deregulation of Twist1 in HCC. Therefore, inactivation of PDGF-D/Twist and activation of miR-106a could be a novel strategy for treatment of HCC.

## MATERIALS AND METHODS

### Cell culture, reagents and antibodies

HepG2 and Huh-7 cells were cultured at 37°C in 5% CO_2_ in Dulbecco's modified Eagle's medium (DMEM; Gibco, Gaithersburg, MD, USA) supplemented with 10% fetal bovine serum. To establish GR cell lines, cells were continuously exposed to increased concentration of gemcitabine for more than 6 months until cells displayed resistance to gemcitabine. MTT [3-(4,5-dimethythiazol-2-yl)-2,5-diphenyl tetrazolium bromide] was purchased from Sigma (St. Louis, Mo). The secondary antibodies and primary antibodies against Snail, Slug, and GAPDH were bought from Santa Cruz Biotechnology (Santa Cruz, CA). Anti-PDGF-D, anti-Twist1, anti-Vimentin, anti-E-cadherin antibodies were obtained from Abcam.

### Cell growth study by MTT assay

The cells (5 × 10^3^) were seeded at equal densities into a 96-well culture plate for overnight incubation. Then, the cells were treated with different concentrations of gemcitabine for 72 hours. MTT assay was performed as described previously [10].

### Wound healing assay

The cells were seeded in 6-well plate until the cells grew to 90–95% confluency. The scratch wound was generated in the surface of the plates with a 20 μl pipette tip. The scratch area was photographed by a microscope at 0 hour and 20 hours.

### Cell attachment and detachment assay

Cell attachment and detachment assays were performed as described previously [54]. Briefly, for attachment assay, 5 × 10^4^ cells per well were seeded in 24-well plates. After 1 h, unattached cells were removed. The attached cells were counted after trypsinization. The data were presented as a percentage of the attached cells compared to total cells. For cell detachment assay, the cells were incubated with 0.05% trypsin for 3 minutes to detach the cells after 24 hours incubation. Then, the detached cells were collected. The remaining cells were counted after detached with 0.25% trypsin. The data were presented as a percentage of the detached cells to total cells.

### Transwell invasion assays

The invasive capacity of cells was performed using Transwell inserts with Matrigel (BD Biosciences). The cells were seeded in a Matrigel-coated chamber. The upper chamber has serum-free media, whereas bottom chamber has complete growth media. After 16 hours of incubation, the upper surfaces of the Transwell chambers were scraped with cotton swabs, and the invaded cells were fixed and stained with Giemsa solution. The stained cells were photographed under a light microscope.

### Reverse transcription-PCR analysis for gene expression

The total RNA from cells was isolated with Trizol (Invitrogen) and purified with RNeasy Mini Kit and RNase-free DNase Set (Qiagen) according to the manufacturer's protocols. The expression of GAPDH was used as internal control. The primers used in the PCR reactions and RT-PCR amplifications were performed as described before [10].

### Protein extraction and western blotting

Cells were harvested and lysed with RIPA buffer (1 × PBS, 1% Nonidet P40, 0.5% sodium deoxycholate, 0.1% SDS, and protease inhibitor cocktail). The protein concentrations were measured using the Bio-Rad protein assay kit (Bio-Rad Laboratories, CA). Immunoblotting was conducted with standard protocols as described previously [55].

### miRNA real-time RT-PCR

The miRNA real-time RT-PCR assay was performed using miR-106a TaqMan MicroRNA Assay Kit (Applied Biosystems). Briefly, ten nanogram of total RNA was reverse transcribed into cDNA and then real-time PCR was performed using specific primers for miR-106a as described previously [56].

### Transfection

Cells were seeded in six-well plates and transfected with different siRNAs or PDGF-D cDNA (Genepharma, Shanghai, China), or miR-106a mimics (Genepharma, Shanghai, China) using Lipofectamine 2000 as described earlier [10]. After the indicated periods of incubation, the cells were subjected to further analysis as presented under the results section.

### miRNA-106a inhibitor tranfection

Cells were seeded in six-well plates and transfected with antisense miR-106a olignucleotide (Genepharma, Shanghai, China) or the nonspecific control using DharmaFect Transfection Reagent (Dharmacon, CO) as described previously [56].

### Luciferase assays

The wild-type and mutant Twist 3′-UTR were amplified by PCR and cloned in pMIR-REPORT (Ambion) with firefly luciferase. A total of 5 × 10^4^ cells treated with control, miR-106a mimics, or miR-106a inhibitors were transfected with wild-type or mutants of Twist 3′ UTR luciferase reporters together with Renilla plasmid. After 48 hours of transfection, the firefly and Renilla luciferase were measured according to the manufacturer's protocols (Promega). The firefly luciferase activities were normalized to Renilla luciferase activities.

### Human HCC samples

The 76 paired samples of human HCC and their matched adjacent non-cancerous tissues were collected at the time of surgery between 2010 and 2012 at the first affiliated hospital to Bengbu Medical College (Anhui, China). The specimens were frozen in liquid nitrogen immediately and stored at –80°C. The study was approved by the Ethics Committee of Bengbu Medical College.

### Histologic sections and immunohistochemistry

Immunohistochemical studies were performed to determine the expression of PDGF-D, Twist in tumors as described before [57].

### Statistical analysis

Values were shown as means± SEM and analyzed using GraphPad Prism 4.0 (Graph pad Software, La Jolla, CA). Statistical comparisons between different groups were performed using Student *t*-test. The R2 statistic was used for detection of correlation between PDGF-D, Twist, and miR-106a in clinical samples. *p* < 0.05 was considered statistically significant.
